# Complete Genome Sequence of Megaplasmid-Bearing Streptococcus salivarius Strain LAB813, Isolated from the Dental Plaque of a Caries-Free Child

**DOI:** 10.1128/MRA.01092-19

**Published:** 2019-10-10

**Authors:** Siew-Ging Gong, Yuki Chan, Céline M. Lévesque

**Affiliations:** a Faculty of Dentistry, University of Toronto, Toronto, Ontario, Canada; b Faculty of Dentistry, The University of Hong Kong, Hong Kong SAR, China; University of Maryland School of Medicine

## Abstract

Streptococcus salivarius strain LAB813 was isolated from the dental plaque biofilm of a caries-free child with healthy oral tissues. We report here the complete genome sequence of *S. salivarius* strain LAB813. This genome consists of a chromosome of 2.2 Mb and a megaplasmid, pSAL813, of 183 kb.

## ANNOUNCEMENT

Streptococcus salivarius is a predominant member of the oral microbiome that persists throughout the human life. It is not known to initiate infections in healthy or immunocompetent individuals. In fact, several reports have indicated that *S. salivarius* plays a positive role in oral and digestive tract ecology. *S. salivarius* may exert its positive impact through effects on the stability of the microbiome, bacterial interference, and/or host interaction (1). *S. salivarius* colonizes the buccal epithelium and is a resident of the tongue dorsum. *S. salivarius* is also able to coaggregate with various oral colonizers (2). Some strains of *S. salivarius* display antimicrobial activity against virulent streptococci (e.g., group A streptococcus [GAS] and group B streptococcus [GBS]) and therefore contribute to the maintenance of oral, pharyngeal, and intestinal health (3). *S. salivarius* has thus emerged as an important source of safe and efficacious probiotics capable of fostering more balanced, health-associated oral microbiota (4). In a screening of *S. salivarius* strains with probiotic potential, our group isolated strain LAB813 from the supragingival plaque collected from the facial and lingual smooth surfaces of the primary maxillary incisors of a caries-free child aged 5 years and 2 months (University of Toronto REB protocol reference number 32740). The plaque sample was plated on mitis-salivarius-bacitracin agar supplemented with 20% sucrose using a spiral plater. Isolate LAB813 was verified as *S. salivarius* by PCR (5) and 16S rRNA gene sequencing (6). Here, we present the complete genome sequence of this strain.

The LAB813 strain was cultivated in a 50-ml volume of brain heart infusion broth at 37°C in air with 5% CO_2_ for 18 h without agitation. Total genomic DNA was extracted using an in-house protocol. Briefly, LAB813 cells were lysed with lysozyme (50 mg/ml at 37°C for 1 h), and proteins were digested with proteinase K (20 mg/ml at 37°C for 15 min) and precipitated with ice-cold potassium acetate buffer and ice-cold isopropanol. The DNA was fished out using a glass pipette and treated with RNase A (10 mg/ml at 37°C for 1 h). DNA was quantified using the Quant-iT PicoGreen double-stranded DNA (dsDNA) assay kit (Thermo Fisher). Whole-genome sequencing was performed using the Pacific Biosciences sequencing technology. The DNA library was prepared following the Pacific Biosciences 20-kb template preparation using the BluePippin size selection system protocol. Qualified genomic DNA was fragmented using the Covaris g-TUBE device and then end repaired to prepare SMRTbell DNA template libraries (with a fragment size of 15 kb to 50 kb) selected using a BluePippin system. Sequencing was performed in a Pacific Biosciences RS II sequencer using the MagBead OneCellPerWell (OCPW) protocol at the Génome Québec Innovation Centre and Canadian Centre for Computational Genomics (McGill University, Québec, Canada). The Pacific Biosciences sequencing using 1 single-molecule real-time (SMRT) cell generated a total of 52,186 raw subreads with an average length of 14,073 bp, with an *N*_50_ value of 24,132 bp. Genome assembly was done using the Hierarchical Genome Assembly Process (HGAP) workflow with default settings (7). The assembled genome had 350× genome coverage. The assembly contained both a complete chromosome of 2,242,557 bp, with a G+C content of 40.1%, and a megaplasmid named pSAL813 of 183,700 bp, with a G+C content of 34.9%. Gene prediction and annotation were performed using RAST (8) and BLASTp (9). A total of 2,101 and 174 protein-coding genes (CDSs), 75 and 0 tRNAs, and 5 and 0 rRNAs were annotated in the chromosome and megaplasmid pSAL813, respectively. The genomic information was analyzed to predict putative bacteriocin gene clusters through the BAGEL4 (10) Web server with default search options. The pipeline predicted a novel multipeptide lantibiotic locus on pSAL813 that is highly similar to the *pld* locus that drives the production of the broad-spectrum bacteriocin pneumolancidin in Streptococcus pneumoniae (11). The megaplasmid also encodes the chromosomal PezTA toxin-antitoxin system found in pneumococci (12). We also identified a chromosomal locus encoding three major fimbrial subunits belonging to the serine-rich repeat proteins family in streptococcal and staphylococcal species and that comprised many repeats of a motif of four amino acid residues (13); we also identified numerous genes encoding enzymes responsible for the glycosylation of the subunits and their transport through the accessory SecA2/Y2 system.

### Data availability.

The complete genome sequence has been deposited in GenBank under the accession numbers CP040803 (megaplasmid pSA813) and CP040804 (chromosome). Raw sequencing reads were deposited in the NCBI Sequence Read Archive (SRA) under accession number SRR9298671 and BioProject number PRJNA546007.
